# Factors That Optimize Reproductive Efficiency in Dairy Herds with an Emphasis on Timed Artificial Insemination Programs

**DOI:** 10.3390/ani11020301

**Published:** 2021-01-25

**Authors:** Carlos Eduardo Cardoso Consentini, Milo Charles Wiltbank, Roberto Sartori

**Affiliations:** 1 Department of Animal Sciences, Luiz de Queiroz College of Agriculture of University of São Paulo (ESALQ/USP), Piracicaba, SP 13418-900, Brazil; carlos.consentini@usp.br; 2 Department of Animal and Dairy Sciences, University of Wisconsin-Madison, Madison, WI 53706, USA; wiltbank@wisc.edu

**Keywords:** cattle, fertility, timed-AI, dairy cows, management, reproductive tools

## Abstract

**Simple Summary:**

Reproductive efficiency is critical for profitability of dairy operations. The first part of this manuscript discusses the key physiology of dairy cows and how to practically manipulate this reproductive physiology to produce timed artificial insemination (TAI) programs with enhanced fertility. In addition, there are other critical factors that also influence reproductive efficiency of dairy herds such as genetics, management of the transition period, and body condition score changes and improve management and facilities to increase cow comfort and reduce health problems. Using optimized TAI protocols combined with enhancing cow/management factors that impact reproductive efficiency generates dairy herd programs with high reproductive efficiency, while improving health and productivity of the herds.

**Abstract:**

Reproductive efficiency is closely tied to the profitability of dairy herds, and therefore successful dairy operations seek to achieve high 21-day pregnancy rates in order to reduce the calving interval and days in milk of the herd. There are various factors that impact reproductive performance, including the specific reproductive management program, body condition score loss and nutritional management, genetics of the cows, and the cow comfort provided by the facilities and management programs. To achieve high 21-day pregnancy rates, the service rate and pregnancy per artificial insemination (P/AI) should be increased. Currently, there are adjustments in timed artificial insemination (TAI) protocols and use of presynchronization programs that can increase P/AI, even to the point that fertility is higher with some TAI programs as compared with AI after standing estrus. Implementation of a systematic reproductive management program that utilizes efficient TAI programs with optimized management strategies can produce high reproductive indexes combined with healthy cows having high milk production termed “the high fertility cycle”. The scientific results that underlie these concepts are presented in this manuscript along with how these ideas can be practically implemented to improve reproductive efficiency on commercial dairy operations.

## 1. Introduction

For decades, genetic selection in dairy cattle was primarily focused on milk production. This genetic selection for production, combined with advances in nutrition, management, facilities, and veterinary programs have generated the modern dairy herds with high milk production (9000 to >12,000 kg of milk in a 305-day period). It has been recognized that primary selection for production lead to cows with poorer reproductive efficiency and health traits [1]. During the last two decades, increased selection for traits linked to reproduction combined with the reliability gains that genomics provided for less heritable traits, such as reproduction, has led to tremendous progress among dairy herds regarding genetic potential for reproduction in the modern dairy cow [2,3,4]. Nevertheless, there are so many factors that affect reproductive efficiency in dairy cattle that a multifaceted approach is required to optimize reproductive performance on high production dairy herds. One approach that has been used with great success on many dairy farms across the globe is to have a systematic reproductive management program that includes timed artificial insemination (TAI) [5,6]. In this review, first, we consider the key physiology that underlies the development of high fertility TAI programs (Section 2) and how this physiology can be practically implemented in TAI programs (Section 3). Specific high-fertility TAI programs are presented in Section 4. Subsequently, key management/cow factors that can alter the efficiency of these reproductive programs are considered (Section 5), and then we conclude with thoughts on practically combining these concepts in programs that optimize reproductive efficiency on dairy herds (Section 6). The goal of this review is to provide scientists, veterinarians, dairy consultants, and dairy producers with up-to-date scientific information on reproductive efficiency in dairy herds that use TAI.

Quantification of reproductive efficiency on dairy farms can be accomplished through a variety of measures. In this review, we primarily use the 21-day pregnancy rate (21 d-PR) because of the utility of this measure for making immediate management decisions on a dairy farm. The 21 d-PR is defined as the percentage of eligible cows that become pregnant every 21 d. The 21 d-PR is most efficiently calculated on a computer. First, the number of eligible cows during each 21-day period must be calculated (i.e., cows past the voluntary waiting period (VWP), that are not pregnant, and not designated as “do not breed”) including whether a cow should be included that is eligible for only a portion of the 21-day period (usually, if eligible for >11 d in a 21-day period they are included). Thus, it would be better to consider “eligible cows” to be “eligible 21-day periods” because a cow can be eligible during multiple 21-d periods before she becomes pregnant. Next, the number of cows that became pregnant during that 21-d period, either due to AI after estrus or TAI, are determined and divided by the number of eligible cows in that 21-day period. Thus, the 21 d-PR can only be determined after definitive pregnancy diagnosis. Two other key measures determine the 21 d-PR on a dairy and should be calculated when evaluating reproductive efficiency on a dairy farm. The 21-day service rate (21 d-SR) is a calculation of the percentage of eligible cows that are serviced (receive AI) during a 21-day period. The 21 d-SR can be calculated immediately after finishing the 21-day period because it does not include determination of whether a cow became pregnant. The final key measure is the pregnancies per artificial insemination (P/AI), inaccurately called conception rate in some circles. The P/AI should be calculated separately for the first AI (first AI P/AI) and second and later AIs (2nd + AIs). This is because programs that yield differences in fertility are generally used for the first vs. later services.

A brief consideration of the link between reproductive efficiency and profitability is appropriate, although this is considered in much greater depth in other manuscripts [7,8,9,10,11,12,13,14]. One key consideration is that cows in the first third of lactation provide greater income over feed cost as compared with cows in the middle or at the end of lactation [15]. In addition, multiparous cows generally have much greater milk production during the first third of their lactation than primiparous cows. Hence, greater milk production per cow per day and efficiency of milk production can be achieved by increasing the percentage of cows in early lactation and the percentage of cows in later lactations (older cows). Thus, one goal of reproductive management programs is to maximize the number of cows that become pregnant early in lactation in order to increase production efficiency and production per cow per day. For instance, [15] reported that a reduction in calving interval of 60 d increased milk production per day (1.51 and 1.11 kg/d) and during entire lactation (~498 and ~366 kg/lactation) in both high-production (12,500 kg in 305 d of lactation) and moderate-production herds (9000 kg in 305 d of lactation), respectively.

An additional key profit generator from efficient reproduction is a reduction in the need for culling high merit cows due to poor reproduction, resulting in either reduced culling or a shift in culling to cows with lower milk production, disease problems such as mastitis, and udder, genetic, or foot and leg issues. Thus, there is an improvement in the overall quality of the herd when reproductive efficiency is improved. Economic benefits may also arise in herds with greater reproductive efficiency due to reduction in reproductive costs, such as costs for semen, reproductive hormones, and veterinary costs such as pregnancy diagnoses, although this will vary with the method used to improve reproductive efficiency [7,11,13]. Finally, as discussed in detail later in this review, greater reproductive efficiency will cause a greater percentage of cows to enter “the high fertility cycle” leading to many benefits in terms of improved health, production, and reproduction [16].

## 2. Five Key Physiologic Factors That Influence Fertility in Timed Artificial Insemination (TAI) Protocols

**Figure 1:**
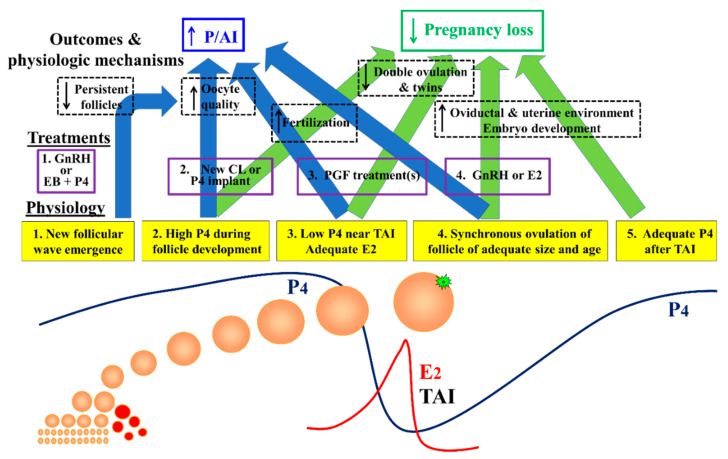
Key physiology (yellow rectangles) that should occur during timed artificial insemination (TAI) protocols in lactating dairy cows. Rectangles with purple lines show common treatments that are used to achieve these results and rectangles with dashed black lines show the mechanisms that produce increased pregnancy per artificial insemination (P/AI) or reduced pregnancy loss in TAI protocols. Corpus luteum (CL), estradiol (E2) benzoate (EB), gonadotropin-releasing hormone (GnRH), progesterone (P4), prostaglandin F2α (PGF).

Protocols for TAI can be broadly divided into two the following pharmacological bases: (1) Ovsynch-type protocols using gonadotropin releasing-hormone (GnRH) and (2) protocols that use estradiol (E2) compounds plus treatment with progesterone (E2/P4 protocols). Regardless of the hormonal combinations, the overall physiological objectives are similar, as summarized in Figure 1. First, the protocol attempts to synchronize emergence of a new follicular wave either by ovulating a dominant follicle after GnRH treatment or by inhibiting gonadotropins after treatment with E2 compounds plus P4 to induce turnover of follicles in the current follicular wave. Second, circulating P4 is maintained at elevated concentrations during development of the new preovulatory follicular wave. Third, efficient regression of the corpus luteum (CL) using prostaglandin F2α (PGF) minimizes P4 and enhances circulating E2 near TAI. Fourth, a follicle with adequate size and age is synchronously ovulated using either GnRH or E2 to correspond with proper scheduling of TAI. Finally, elevated and consistent circulating P4 is maintained from properly functioning CL generated after the final ovulation.

Synchronized emergence of a new follicular wave minimizes development of persistent follicles during the protocol (Figure 1). Previous studies have shown that ovulation of follicles that have prolonged periods of follicular dominance can dramatically reduce fertility of lactating dairy cows [17]. Prolonged dominance may reduce fertility by decreasing oocyte quality, possibly by allowing premature meiotic resumption due to high luteinizing hormone (LH) pulse frequency [18]. Although oocytes from these persistent follicles appear to be efficiently fertilized, the embryo stops developing prior to the blastocyst stage [19]. In a study that evaluated ovarian dynamics during an E2/P4 TAI protocol, cows without follicle wave emergence at the beginning of the protocol that subsequently ovulated persistent follicles at the end of the protocol had lower P/AI compared to cows that had emergence of a new follicular wave (21 vs. 43%) [17]. Similarly, in GnRH-based protocols, P/AI was greater in cows that ovulated follicles of intermediary size (15–19 mm, 47%) as compared with those ovulating smaller (<14 mm, 36%) or larger (>20 mm, 38%) follicles [20]. Thus, optimizing the follicle size and oocyte quality near TAI depends on the efficiency of the strategy used to initiate emergence of a new follicular wave at the beginning of the protocol.

Secondly, circulating P4 concentrations during preovulatory follicle development have dramatic effects on the subsequent fertility of high-producing lactating dairy cows. Lower circulating P4 during follicular growth, either due to an anovulatory condition [21] or due to the higher catabolism of this hormone in high-producing cows [22,23], is associated with greater pulsatility of LH, which can result in premature resumption of oocyte meiosis and germinal vesicle breakdown, decreasing oocyte quality, and consequently fertility [18,24]. A study by [25] reported that cows yielding over 40 kg/d of milk that were superstimulated to produce multiple ovulations during the first follicular wave (low P4 during follicle growth) had a greater percentage of degenerate embryos (23.5%) as compared with cows superovulated during the first follicular wave but with P4 supplementation (7.1%) or those superovulated during the second follicular wave (3.9%). Moreover, the percentage of transferable embryos was much greater after superovulation during the second follicular wave (88.5%) and the first wave with supplementary P4 (78.6%) as compared with superovulation during the first follicular wave (55.9%). An elegant study evaluated the effect of circulating P4 concentration on embryo quality of cows synchronized and with single ovulation [26], in which the ovulatory follicle developed under a higher or lower circulating P4 milieu. Although fertilization was similar (78% on average), the percentage of grade 1 and 2 embryos (high quality embryos) was greater for cows ovulating follicles that developed under higher P4 (86.5%) than follicles that developed under lower P4 (61.5%). Moreover, cows with higher circulating P4 had fewer degenerate embryos (8.1%) than cows with lower circulating P4 (34.6%).

**Figure 2:**
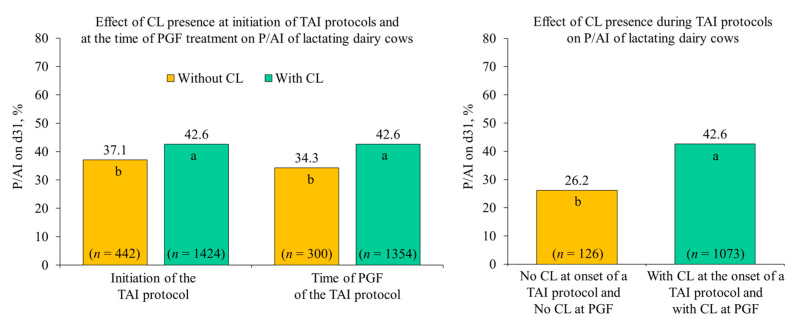
Effect of the presence of corpus luteum (CL) during timed artificial inseminations (TAI) protocols on pregnancy per AI (P/AI) of lactating dairy cows. ^a,b^ Different letters represent differences (*p* < 0.05). Prostaglandin F2α (PGF). Data from 3 experiments from our lab in which cows were submitted to TAI protocols initiated with progesterone implant and estradiol benzoate, GnRH, or both [30].

Many studies have reported greater P/AI when cows were submitted to TAI programs in which a CL was present or the P4 milieu during follicle development was high [27,28,29]. In a compilation of data from studies of our laboratory [30] using P4-based protocols, that started with estradiol benzoate (EB), GnRH, or both, the presence of CL at the beginning of TAI protocols or at the time of PGF increased P/AI by 15–24% (Figure 2), and the best fertility was achieved when CL was present at both times of the protocol (Figure 2).

Another important aspect of circulating P4 concentration during TAI programs is related to double/multiple ovulation and twinning. Double ovulation is more frequent when there is low circulating P4 during the protocol [31,32] and in cows with higher milk yield [33,34]. Another factor that influences double ovulation is parity, in which multiple ovulations have been described to be more frequent in multiparous compared to primiparous cows [35], and this can be explained by the greater milk production in multiparous cows. Double ovulation in dairy cattle is undesirable because it increases the incidence of twin pregnancies [36], which are associated with calving problems, calf mortality, freemartins, and problems with calf development. Moreover, twinning is associated with greater pregnancy loss after 30 d of pregnancy [37,38]. Thus, during preovulatory follicle development, increasing circulating P4 optimizes follicle size and oocyte quality and also can decreases development of co-dominant follicles, multiple ovulation, and twins; this effect may decrease pregnancy loss.

**Figure 3:**
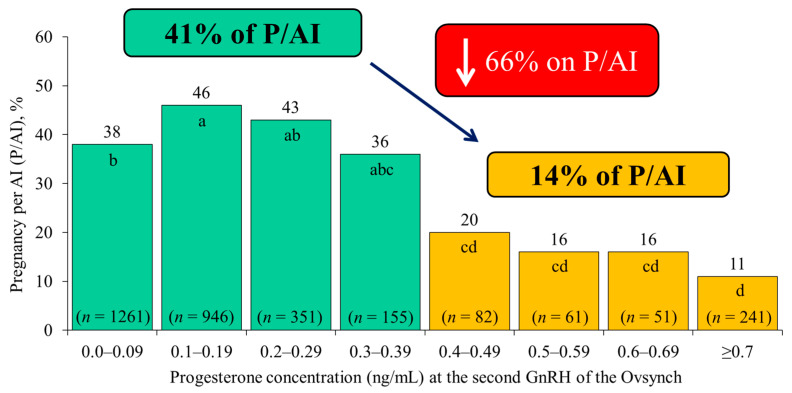
Pregnancy per AI (P/AI) in lactating dairy cows in relation to progesterone concentration at the time of the second gonadotropin releasing-hormone (GnRH) of the Ovsynch protocol. ^a,b,c,d^ Different letters represent differences (*p* < 0.05). From [6].

The third key physiologic outcome to achieve during TAI programs is to efficiently regress the CL, having minimal circulating P4 near TAI. Many studies have reported a relationship between circulating P4 concentrations near TAI and ovulation or fertility [17,20,39,40,41,42] with even small concentrations of P4 near TAI producing dramatic decreases in fertility. For example, in a large data set compiled by [6], there was a 66% relative decrease in P/AI for cows with P4 ≥ 0.4 ng/mL (14%, 161/435) as compared with cows with P4 < 0.4 ng/mL (41%, 1125/2713) at the time of the second GnRH treatment (G2) during the Ovsynch protocol (Figure 3). This outcome is likely to be due to the negative effects of residual P4 on ovulation at the end of a TAI protocol, and on gamete transport [43], hampering fertilization efficiency (Figure 1).

This residual P4 near AI is due to a lack of complete luteolysis after the PGF treatment during the protocols, which may occur in 13 to 44% of cows [44,45], and is more problematic when young CL are present at the time of PGF, due to their lower responsiveness to a single treatment with PGF [46]. Therefore, new strategies have been used in TAI programs to overcome the issue of incomplete CL regression at the end of the protocol, and those strategies are discussed later in this manuscript. 

The fourth key point is related to optimal size and synchronized ovulation of the follicle in relation to TAI. A more optimal size will result in greater E2 concentrations prior to TAI resulting in greater expression of estrus at the end of TAI protocols. In general, cows that express estrus before TAI achieve greater P/AI, in both Ovsynch-type [47] and E2/P4-based protocols [48]. Another positive effect of expression of estrus is a decrease in pregnancy loss, as reported in a study with 5430 cows, in which cows expressing estrus had ~28% lower pregnancy loss than cows not expressing estrus [48]. Cows expressing estrus may also have greater fertility due to greater likelihood of ovulation [49], although an analysis of only cows that ovulated to an E2/P4 protocol still showed an increase in fertility in cows expressing estrus [48]. Similarly, in cows synchronized with GnRH-based protocols, estrus is related to circulating E2, which is greater for cows ovulating larger follicles at the end of the protocol [20], and higher circulating E2 before AI is also associated with greater fertility [50]. Adequate circulating E2 prior to AI is associated with a differential expression of genes in the endometrium and conceptus, likely producing conditions that are favorable to pregnancy [51], and gamete transport [52]. Thus, ovulation of a more optimal size of follicle will result in greater circulating E2 during proestrus and greater expression of estrus. Use of different strategies to induce ovulation can also result in more synchronized ovulation in relation to TAI and this may help fertility.

Finally, the absolute requirement for P4 (or the CL hormone) in pregnancy maintenance was demonstrated over 100 years ago [53,54]. The numerous studies investigating whether P4 supplementation increased fertility in lactating dairy cows starting in the 1950s [55,56], until today, have been less definitive [17,57,58,59]. Among 30 trials that we evaluated for a review manuscript [43], the vast majority (25/30) showed a numeric improvement in fertility with P4 supplementation, but only six of these trials showed significance (*p* < 0.05). Only two [58,60] of these trials that found significance had groups of more than 100 animals per treatment.

The most extensive trials to increase P4 have been done by inducing formation of an accessory CL with hCG or GnRH treatment. When hCG or GnRH is administered on Day 5 after AI, there is generally formation of an accessory CL and increased P4 during the mid-luteal phase [61,62]. For example, we observed 93% ovulation after treatment with 3300 IU of hCG on Day 5 with an increase in circulating P4 by 3 d after hCG treatment from Day 8 until 16 of the cycle [63]. We performed a meta-analysis of 10 previous trials that analyzed the effect of hCG on fertility in a total of 4397 lactating cows [58]. There was a modest (*p* = 0.04) increase of 3% comparing hCG (37.0%, 808/2184) to control cows (34.0%, 752/2213). On the basis of these results, we designed a manipulative study that included data from 2979 lactating dairy cows on six commercial dairies [58]. Treatment with hCG 5 d after AI increased (*p* = 0.01) fertility by 3.5% from 37.3% in controls (566/1519) to 40.8% in hCG-treated cows (596/1460). A surprising observation was that all of the effect of hCG on fertility was due to a dramatic increase in primiparous cows (39.5%, 215/544 control primiparous and 49.7%, 266/535 hCG-treated primiparous) with no change due to hCG treatment in multiparous cows (36.0%, 351/975 control multiparous and 35.7%, 330/925 hCG-treated multiparous). A more recent meta-analysis [59], evaluating about 18,000 cows per treatment group, reported that buserelin (>10 µg) or hCG (>2500 IU) increased P/AI in primiparous cows, particularly, if they had lower fertility (<45% P/AI). Although it is clear that P4 can alter many aspects of endometrial gene expression, uterine histotroph, and increased embryo elongation [64], none of these experiments provide definitive evidence for the mechanisms causing the differences between parities in the fertility effects of hCG. Further research is clearly needed to clarify whether the timing of the P4 increase or other factors can explain the relatively low effect of hCG on fertility and the unexpected parity influence on the hCG effect, or the inconclusive effects of other strategies for P4 supplementation post AI on P/AI in dairy cows.

## 3. Hormonal Strategies to Improve TAI Protocols

### 3.1. Hormonal Strategies to Initiate TAI Protocols

There are two main strategies used to initiate TAI protocols and to promote a new follicular wave emergence. The first one aims to synchronize emergence of a new follicular wave by causing atresia of the follicles present in the ovaries due to a negative feedback in follicle stimulating-hormone (FSH) and LH, promoted by a combination of an increase in circulating E2 (from an E2 ester) and P4 (from intravaginal P4 implants, IVP). This is the physiologic basis for initiation of E2/P4-based protocols. The second strategy, which is the basis for Ovsynch-type protocols, stimulates emergence of a new follicular wave by inducing ovulation of a dominant follicle by exogenous GnRH treatment.

The most used E2 ester along with P4 implants on Day 0 (d0) of TAI protocols is EB using a dose of 2 mg. However, this strategy did not properly synchronize emergence of a new follicular wave in more than 25% of lactating dairy cows [17]. Another study reported 24.2% of cows ovulating a persistent follicle at the end of a protocol starting with EB, GnRH, and a P4 implant [65]. Therefore, this issue can impair fertility considering that older/persistent follicles may ovulate overstimulated oocytes, and therefore result in poorer embryo development in lactating dairy cows.

Studies from our laboratory have focused on strategies to initiate TAI protocols that improve synchronization rates and fertility. In one of those studies [17], increasing the dose of EB to 3 mg did not improve synchronization of emergence of a new follicle wave as compared with treatment with 2 mg (71.4 vs. 81.6%, respectively). Moreover, initiating the protocol with EB plus P4 implants in the presence of young (3 d after a GnRH treatment) or dominant follicles (7 d after GnRH) produced similar wave emergence efficiency (78.7 vs. 82.3%). The overall synchronization rate (follicular wave emergence at the beginning and ovulation at the end) for these traditional E2/P4-based protocols was 32 to 60% in studies from our lab, and P/AI was much greater for synchronized cows than cows that were not properly synchronized (61.3 vs. 15.7%) [17].

Another potential negative factor in P4-based TAI protocols that start with E2 protocols is that treatment with EB at the beginning is associated with a greater incidence of luteolysis between d0 and the time of PGF treatment, decreasing the percentage of cows with CL and the number of CL at PGF, which is related to lower circulating P4 during development of the preovulatory follicle [29,66], compromising fertility. About ~40% of the cows that had a CL present on d0 underwent CL regression between d0 and PGF when EB treatment was at the beginning of a TAI protocol [17,29,66]. Table 1 shows a compilation of data from four studies that compared treatment with EB vs. GnRH or EB vs. EB plus GnRH at the beginning of TAI protocols. Treatment with GnRH increased (22.2%) the percentage of cows with CL at the time of PGF, indicating an increase in circulating P4 during the protocol (Table 1).

The objective of TAI protocols that begin with GnRH is to induce ovulation, resulting in emergence of a new follicular wave and increasing circulating P4 during preovulatory follicle development. Ovulation to GnRH increased circulating P4 at PGF in multiple studies [41,65,69] and increased P/AI [69,70]. Ovulation to GnRH primarily increases P/AI in cows initiating the protocol without CL or with low circulating P4 [66,69,70].

Since ovulation after d0 is associated with greater circulating P4 during follicle development and greater P/AI, optimized TAI programs seek to maximize this response. One strategy is to use presynchronization strategies. Another approach to increase ovulation after d0 of a FTAI protocol is related to the dose and analogue of GnRH. When increased the dose of gonadorelin acetate from 100 to 200 µg [71], there was a greater LH peak, and this was particularly important in cows with greater circulating P4, due to an inhibitory effect of P4 on the GnRH-induced LH peak. In fact, in a study using nonlactating Holstein cows, the dose of 100 µg of gonadorelin induced ovulation in only 58.1% of cows with a 7-day-old CL present compared to 95.5% ovulation in cows without a CL [72].

When comparing two analogues of GnRH, studies from our laboratory [73] have shown that 100 µg gonadorelin acetate produced a lower LH peak compared to 10 µg buserelin acetate in Nelore (*Bos indicus*) heifers (5.4 vs. 11.7 ng/mL) and cows (3.4 vs. 6.9 ng/mL) on Day 7 of the estrous cycle. When the dose of these two analogues was doubled, buserelin increased the LH peak in heifers (11.7 vs. 23.2 ng/mL) and cows (6.9 vs. 13.2 ng/mL), whereas the double dose of gonadorelin only increased the LH peak in cows (3.4 vs. 6.3 ng/mL) but not in heifers (5.4 vs. 5.2 ng/mL). Considering the main effects of the study, buserelin induced a greater LH peak and ovulation than gonadorelin [73]. Other studies have reported greater efficiency of buserelin and lecirelin than gonadorelin [74,75].

Table 2 presents fertility data of studies that compared protocols initiated only with EB vs. GnRH alone or EB plus GnRH, all associated with insertion of a P4 implant. There was greater P/AI (6.1 absolute percentage increase, on average, ranging from 4.5 to 9.5) and 18.5% (relative P/AI) in protocols initiated with GnRH or GnRH plus EB compared to those initiated only with EB. Therefore, it is recommended that TAI protocols in lactating dairy cows should be initiated with GnRH instead of EB, or at least a GnRH treatment should be included at the beginning of the protocol. In addition, doubling the dose of GnRH at the beginning of a TAI protocol may be advantageous to increase the ovulatory response, especially in cows expected to have a CL on d0.

### 3.2. Intravaginal P4 Implants during TAI Protocols

Although intravaginal P4 implants may be used to improve fertility during TAI protocols, it should be noted that P4 implants do not increase circulating P4 in lactating dairy cows compared to the concentrations that are achieved in cows with an active CL. For example, in the study by [77], circulating P4 on d7 and d14 of an estrous cycle in lactating dairy cows was 2.1 and 4.2 ng/mL, respectively. In contrast, in a study from our laboratory, when comparing two commercial P4 devices (1.9 and 2.0 g of P4) in postpartum cows without CL and producing 40.0 kg of milk per day, the peak of circulating P4 was similar between devices (1.6 ng/mL) and the mean P4 during Day 9 of insertion was 0.85 ng/mL (unpublished data). Studies by [27,28] reported greater circulating P4 in cows with a CL during the protocol compared to those without CL that were supplemented with two P4 devices (1.38 g), even though P4 supplementation increased circulating P4 to 1.9 ng/mL. Therefore, TAI protocols can be improved by increasing the proportion of cows that initiate the protocol with a CL, either by decreasing anovulatory conditions or by using presynchronization programs.

A study [28], with more than 600 cows per group, compared cows initiating the Ovsynch protocol with a CL present on d0 to cows without CL on d0 supplemented or not with two P4 implants with 1.38 g of P4, each. Cows without CL at the beginning of the protocol had the lowest fertility (31.3%), but P/AI on d32 did not differ between cows with CL and those without CL, but supplemented with P4 (38.4 and 42.2%, respectively). In a study with ~160 cows per group, using E2/P4-based TAI protocols and analyzing only cows that ovulated at the end of the TAI protocol, cows treated with two P4 implants tended to have greater P/AI on d60 compared to cows receiving only one implant (48.1 vs. 37.7%) [78].

In a meta-analysis done in 2015 [79], with 25 studies and more than 16,000 cows supplemented or not with one P4 implant, there was a greater risk of pregnancy on d32 and d60 in P4-supplemented cows, but mainly in cows without CL at the beginning of the TAI protocol. Moreover, P4 supplementation tended to reduce pregnancy loss. It is important to mention that in the meta-analysis, cows inseminated in estrus during the TAI program had no benefit from P4 supplementation [79].

Therefore, besides the need for P4 implants in E2/P4-based protocols, Ovsynch-type protocols may also benefit by the addition of P4 implants due to better synchronization of wave emergence, improved oocyte quality, improved luteolysis after single PGF treatments, and reduced double ovulation and twins.

### 3.3. Additional PGF Treatment during TAI Protocols

Complete luteolysis is essential for optimal fertility during TAI protocols (Figure 3). Therefore, the following two strategies have been used to achieve this outcome: (1) increasing the dose of PGF [45,69], and (2) adding a second treatment with PGF, in general, 24 h after the first one [39,41,44,45,80,81].

Increasing the dose of cloprostenol sodium from 500 to 750 µg during a double-Ovsynch program increased the percentage of multiparous cows with complete luteolysis (87.7 vs. 79.2%) but not primiparous cows (92.8 vs. 89.7%) [69]. Interestingly, doubling the dose of dinoprost tromethamine from 25 to 50 mg during the Ovsynch protocol [45] did not increase the percentage of cows with complete luteolysis (88 vs. 88%) and did not increase P/AI (30.2 vs. 32.4%). However, two treatments with PGF 24 h apart increased the proportion of cows with complete luteolysis (88 vs. 94%) and increased P/AI (30.2 vs. 35.4%).

A meta-analysis with seven studies, 5356 cows analyzed for P/AI and 1856 cows analyzed for luteolysis, evaluated the effect of an additional treatment with PGF during the Ovsynch protocol [82]. This analysis reported that 11.6% (6 to 14%) more cows had complete luteolysis when two PGF treatments were employed, and there was a 4.6% increase in P/AI (13.5% relative P/AI, Table 3).

### 3.4. Strategies to Induce Final Ovulation in TAI Programs

Synchronized ovulation of the dominant follicle at the end of TAI protocols can be induced by E2 esters, such as EB or E2 cypionate (EC) [29], or with GnRH, as in Ovsynch [83], potentially altering fertility. The use of EC is convenient because it can be administered concomitant with the final PGF of the protocol or P4 implant withdrawal [29]. However, the timing of ovulation induced by EC is more variable than when GnRH is used [84,85]. On the other hand, when GnRH is used at the end of TAI protocols, optimal fertility is only achieved if cattle are handled one additional time. Moreover, expression of estrus is reduced.

Our group designed a large experiment to compare fertility in response to different inducers of ovulation that were administered at times that were considered to be ideal for fertility in TAI protocols [86]. A protocol for synchronization of ovulation was initiated (d0) after a novel presynchronization with 16.8 µg of buserelin acetate and insertion of a 2.0 g P4 implant, followed by a PGF treatment on d6, and a second PGF on d7, concomitant with the removal of the P4 implant. In Group EC, cows received 1.0 mg EC on d7 as an inducer of ovulation. In Group G, cows received 8.4 µg GnRH at 56 h after the first PGF (16 h before TAI). In Group EC/G, cows received both EC and GnRH. The TAI was performed on d9 (48 h after P4 withdrawal) in all experimental treatments, and pregnancy diagnosis was performed 31 and 60 d after TAI.

Our hypothesis was that the EC/G group would have the greatest P/AI, due to a more synchronized ovulation in response to GnRH plus greater estrus expression because of E2 supplementation, but this idea was not supported. Pregnancy per AI on d31 was not different among the strategies to induce final ovulation (~43%). Other studies that compared EC to GnRH as ovulation inducers also reported similar fertility between treatments [84,85]. Although pregnancy loss tended (*p* = 0.09) to be greater in cows receiving only GnRH as ovulation inducer (Table 4), there was no detectable difference (*p* = 0.54) in P/AI on d60 among treatments. Additionally, when the two groups that received EC (EC and EC/G) were combined, there was lower pregnancy loss compared to cows receiving only GnRH [11.2 (21/188) vs. 19.8 (17/86), *p* = 0.05). The potential for greater pregnancy loss in group G is rationally explained by a lower circulating E2 concentration during proestrus. This could alter the oviductal or uterine environment, potentially increasing pregnancy loss, similar to the increased pregnancy loss observed in cows that did not express estrus during E2/P4 protocols [48]. Therefore, the traditional strategies to induce final ovulation in TAI programs such as GnRH 16 h and EC 48 h before TAI, in general, provide good overall fertility and can be chosen by dairy operations according to management and costs.

## 4. Fertility Programs: First AI and Resynch Protocols with Improved Fertility

### 4.1. Protocols for First TAI That Produce Better Fertility than AI to Estrus

**Figure 4:**
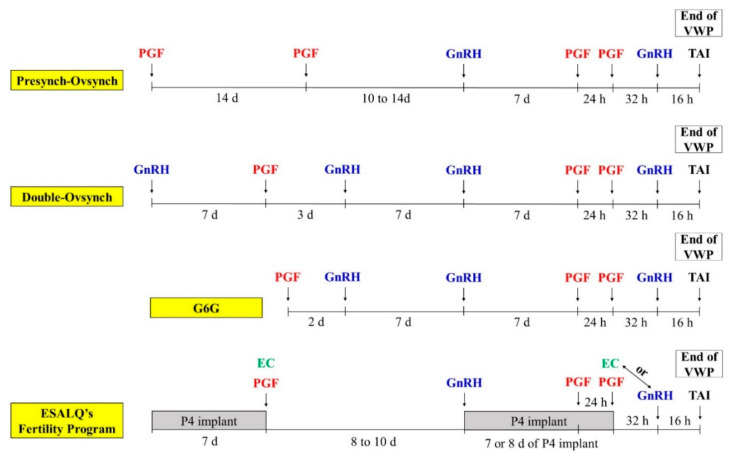
Hormonal treatments used in four fertility programs that are discussed in the text. These programs can be used to achieve 100% service rate and improved pregnancy per artificial insemination compared to the first postpartum timed artificial insemination (TAI) in lactating dairy cows. Voluntary waiting period (VWP), gonadotropin-releasing hormone (GnRH), prostaglandin F2α (PGF), progesterone (P4), and estradiol cypionate (EC).

It is well known that one of the greatest benefits of reproductive programs using TAI is the increase in service rate. In addition, optimized TAI protocols can provide extra benefits associated with greater P/AI when compared to programs for AI after estrus synchronization/detection. Thus, those optimized TAI protocols have been termed “fertility programs” [5,6] and four of these programs that are discussed below are presented in Figure 4.

Fertility programs use many of the principles and strategies discussed above to optimize synchronization, ovarian dynamics, and hormonal environment during TAI protocols. A critical aspect of these programs is the use of a presynchronization strategy in order to ensure that most of the cows initiate the breeding protocol (initiated with GnRH) at an ideal stage of the estrous cycle (6–8 d of the cycle), in which cows have an approximately seven-day-old CL and the first wave dominant follicle that will be responsive to the first GnRH. As previously discussed, increasing ovulation response to the first GnRH will increase the percentage of cows with synchronized emergence of a follicular wave. Causing a new ovulation in the presence of a seven-day-old CL results in cows with two CL throughout the protocol, thus, increasing circulating P4 during the development of the preovulatory follicle. Due to the longer duration of these fertility programs, in general, they are used exclusively for the first postpartum AI, especially because presynchronization strategies can be applied before the end of the VWP, resulting in no delay in receiving the first AI.

One of the earlier presynchronization programs developed was based on PGF administrations, known as the Presynch-Ovsynch protocol (PO) [87]. The PO is based on a PGF treatment, followed by a second PGF 14 days later, and initiation of an Ovsynch-type protocol 10 to 14 days after the second PGF [87,88,89,90]. In general, PO increased fertility compared to Ovsynch [88,89]. In the study by [87] P/AI was 42.8 (113/264) vs. 29.4% (80/272) for PO and Ovsynch, respectively, but it should be mentioned that PO only increased fertility in cyclic cows. Therefore, one disadvantage of the PO is that it is only effective in cyclic cows. Thus, if the percentage of cows that are anovular is high in the herd during early lactation, other strategies that induce ovulation during the presynchronization are likely to be more efficient. In addition, PO does not precisely synchronize cows to be in the ideal day of the cycle on d0 of Ovsynch, because it is based on inducing cows to be in estrus with variable timing after PGF treatments [90,91]. This may produce a less than ideal timing for starting the Ovsynch protocol (6–8 d). A final aspect to be considered is whether cows that are observed in estrus during the Presynch-Oyvsynch protocol should receive AI. The percentage of cows detected in estrus after the second PGF can be over 50% [90,91,92], and it is common for dairy operations to inseminate those cows. Although the cows are being inseminated earlier postpartum, fertility is generally lower compared to not breeding cows that show estrus and inseminating all cows at the TAI after PO [93]. Thus, if the herd submits cows to TAI at the end of the PO, this can be considered to be a fertility program, even with the considerations regarding anovulatory condition and accuracy of the presynchronization with PGF.

The second fertility program presented in this manuscript is the G6G or G7G. Commonly, cows receive a PGF treatment and 2 days later a GnRH treatment, 6 or 7 days before initiating the Ovsynch protocol. Therefore, the G6G/G7G should increase the percentage of cows at the ideal stage of the estrous cycle to initiate Ovsynch. In addition, the inclusion of GnRH during the presynchronization may benefit anovular cows. The G6G/G7G is commonly used in commercial dairy herds and several studies have tested this strategy [44,94,95]. One of them [50] reported greater ovulation to the first GnRH (85 vs. 54%), greater response to PGF (96 vs. 69%), better synchronization rate (92 vs. 69%), and greater P/AI (50 vs. 27%) in cows submitted to G6G compared to Ovsynch initiated at random days of the estrous cycle.

The third fertility program is the double-Ovsynch program (DO) [96]. The DO was developed to optimize the response to hormonal treatments during the breeding Ovsynch protocol, increasing synchronization and the hormonal milieu during follicle development. In the study by [96], when compared to PO, DO decreased the percentage of cows with P4 < 1.0 ng/mL (9.4 vs. 33.3%) at the time of the first GnRH of the breeding Ovsynch, increased circulating P4 at PGF (4.2 vs. 3.2 ng/mL), and increased P/AI (49.7 vs. 41.7%). In a study, which compared the DO and the PO, in relation to circulating P4 concentrations and ovulation to GnRH treatments [97], 94% of the cows in the DO had CL at the time of the first GnRH compared to 68% of the cows in the PO. Moreover, ovulation to the first GnRH was greater in the DO (80%) compared to the PO (69.9%), and the percentage of cows with P4 ≥ 1.0 ng/mL at PGF was greater in the DO than the PO (88 vs. 76%). Another study, with ~1700 cows, which compared the DO and the PO, reported a greater uniformity of intermediary P4 concentrations at first GnRH treatment of the breeding Ovsynch protocol in cows submitted to the DO, and only ~6% of the cows had P4 < 0.5 ng/mL at the beginning of the breeding protocol compared to ~25% in the PO program [98]. There was a clear benefit of the DO to anovular cows and greater incidence of ovulation in response to the presynchronization treatments. In this study, P/AI was greater with the DO (46.3 vs. 36.8%), with a greater effect in primiparous (52.5 vs. 42.3%, *p* = 0.02) than multiparous (40.3 vs. 34.3%, *p* = 0.07) cows. This increased fertility in cows synchronized with DO has also been described in an elegant study that submitted cows to TAI after the DO compared to a protocol designed to increase expression of estrus, with all cows being inseminated at similar days in milk (DIM) (~77 DIM) [99]. Cows in the DO group for first AI had 100% service rate compared to 77.5% in cows bred to estrus. There was also an increase in P/AI from 38.6 to 49.0%, and a 27% relative increase in P/AI when the DO was used (Table 5). Due to the increase in both service rate and P/AI with DO, there was more than a 50% increase in the 21-day PR (Table 5).

Recent experiments from our laboratory used a novel presynchronization strategy prior to breeding protocols that are initiated with GnRH [86]. The presynchronization was based on E2 and P4, using an intravaginal P4 implant that was removed after 7 d. At the time of P4 implant withdrawal, cows were treated with PGF and EC to induce estrus and ovulation. Eight to 10 d later, the cows were treated with GnRH to initiate the first postpartum TAI protocol and an intravaginal P4 implant was inserted and kept for 7 or 8 d. One d before and at the time of P4 implant removal, PGF treatments were given. Ovulation at the end of the protocol was synchronized with EC (given at the time of P4 implant withdrawal), GnRH (given 16 h prior to FTAI), or both. The P/AI varied from 32 to 58% among six farms, with an overall P/AI of 43%. Compared to regular TAI protocols that were initiated at random stages of the estrous cycle, the fertility program increased P/AI (59.9 vs. 43.9% [*n* = 663] and 46.4 vs. 30.1% [*n* = 416], for data set 1 and 2, respectively).

Therefore, use of TAI protocols can increase SR by allowing AI of all cows without the need for detection of estrus. Use of more optimized TAI protocols have the advantage of increasing P/AI compared to AI to detected estrus and thereby can dramatically increase the percentage of cows that become pregnant during the first week after the end of the VWP.

### 4.2. Reinsemination Strategies: Reducing the Interval between AI and Optimizing Fertility

After submitting cows to the first postpartum AI, it is imperative to identify nonpregnant cows as soon as possible and to reinseminate them as early as possible. The most common strategies to reinseminate nonpregnant cows in dairy herds are either detecting estrus or inseminating them using TAI programs after nonpregnant diagnosis (NPD). Several strategies were developed to increase reinsemination rates of cows by detection of estrus [100,101,102]. In general, it was concluded that, in herds with relatively high estrus detection rates and good fertility at AI by estrus, the reproductive performance can be similar to those using or including TAI. However, the herd will always have less control of the interval between inseminations, which can be longer than when submitting cows to TAI Resynch programs.

Regarding resynchronization of ovulation to reinseminate cows using TAI, many studies have been carried out to either understand the physiology or to improve the efficiency of resynchronization (Resynch) programs. It is common to initiate the resynchronization protocol at the time of the NPD. However, there are strategies that initiate the TAI protocol before NPD, which include presynchronization protocols or use P4 supplementation [40,41,102,103,104,105,106,107,108,109].

Initiating the resynchronization protocol at NPD diagnosis either at d32 or d39 after a previous AI did not differ in terms of P/AI [40,110]. However, presynchronization with a GnRH treatment 7 days before the onset of the protocol improved fertility [40,110], and P4 supplementation increased P/AI, especially in cows without CL or with P4 < 1.0 ng/mL at the beginning of the resynchronization protocol [109,110].

In one study [111], cows received a GnRH treatment 32 days after a previous AI, and 7 days later (time of NPD, 39 d after a previous AI), and were divided into three groups as follows: (1) no CL, CL < 15 mm or cystic (cows initiated an Ovsynch + P4 protocol and were inseminated on d49); (2) cows with CL > 15 mm (cows received the final treatments of the Ovsynch, with PGF at NPD, GnRH 16 h before TAI, and were inseminated on d42); (3) no CL, CL < 15 mm or cystic (cows received a GnRH on d39 and had the Ovsynch initiated on d46, being reinseminated 56 days after the previous AI). Cows in suboptimal conditions for fertility, when received an Ovsycnh + P4 protocol or a presynchronization with a GnRH treatment before the Ovsynch, had their fertility restored [111]. However, the authors did not observe improved performance for Ovsynch + P4 (cows reinseminated earlier) as compared with cows reinseminated after the presynch + Ovsynch program. It should be mentioned that all protocols in that study included only one PGF treatment at the end.

Another study [108] evaluated whether the interbreeding interval could be shortened by using a shortened resynchronization strategy that used treatments based on ovarian structures found at the D32 NPD. Thus, two strategies were compared as follows: (1) Resynch-32, a conventional Resynch-32 (with only one PGF) and TAI at d42 after previous AI and (2) shortened Resynch. Cows were evaluated for ovarian structures at the D32 NPD, cows with a CL ≥ 15 mm and a follicle ≥ 10 mm were treated with two PGF, 24 h apart, and GnRH 16 h before TAI, and received TAI at d35 after previous AI or cows that did not have a CL > 15 mm at NPD were treated with an Ovsynch + P4 protocol that included two PGF and were inseminated on d42 after previous AI. The shortened Resynch strategy reduced time to pregnancy by 16 days (79 vs. 95 days), improved the likelihood of achieving pregnancy (1.18 hazard ratio), but did not affect overall P/AI (33.9 vs. 31.0%) compared to the conventional Resynch-32 protocol [108]. This could be an efficacious Resynch program for herds that use ultrasound for NPD at d32 after AI.

A more recent study [109] compared Resynch-32 (with two PGF treatments) to a management strategy in which nonpregnant cows with CL at NPD received two PGF 24 h apart, and GnRH 16 before TAI (on Day 35), while cows without CL at NPD were enrolled in a Ovsynch + P4 protocol, with two PGF at the end, and were reinseminated 42 days after a previous AI. The authors reported that the management strategy that evaluated ovarian structures to enroll cows in the selected Resynch strategies was a viable alternative to reduce inter-insemination intervals [109].

Most of the studies discussed above included detection of estrus as part of their management strategy. Therefore, the efficacy of the TAI Resynch programs can be confounded by differences in detection of estrus before NPD and the potential that fertility would be different in cows that were detected in estrus compared to if all the cows had entered the Resynch TAI program.

**Figure 5:**
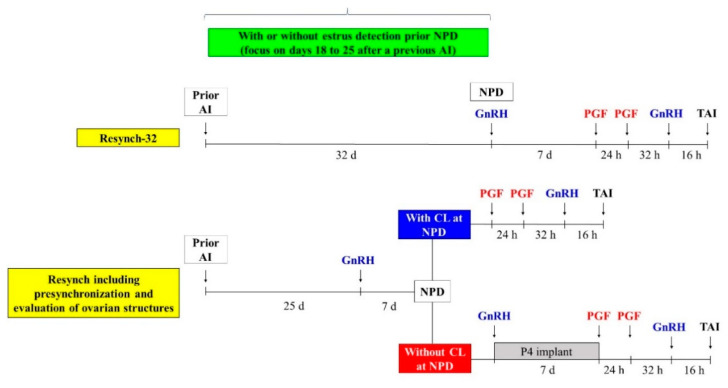
Schematic representation of two commonly used reinsemination programs, designed to reduce the interval between timed artificial insemination (TAI) and optimize fertility. The timing of nonpregnancy diagnosis (NPD) is the same in both protocols (32 days after AI) and is a commonly used timing in herds using transrectal ultrasound for pregnancy diagnosis. The upper strategy is Resynch-32, while the lower strategy is designed to produce earlier TAI in most non-pregnant cows and to improve fertility to the TAI by using a corpus luteum (CL) evaluation at the NPD ultrasound. Gonadotropin-releasing hormone (GnRH), prostaglandin F2α (PGF), progesterone (P4).

Thus, dairy operations have several options for managing their reinsemination program including using strategies that utilize or do not utilize detection of estrus and TAI programs that reduce the interval between inseminations. In addition, strategies can be used that optimize fertility by identifying cows that were not properly synchronized by the Resynch strategy. Figure 5 shows two common Resynch strategies. The upper strategy is the classical Resynch-32 strategy with Ovsynch initiated at the NPD at d32 after previous AI and TAI done at d42 after previous AI. The lower panel shows a more aggressive Resynch strategy using GnRH treatment at d25 after previous AI and 1 week later (D32) using ultrasound for NPD and to perform a “CL check”. Cows with a CL continue the Ovsynch protocol with TAI at d35 after previous AI (80–85% of non-pregnant cows). Cows without a CL at the NPD would have very low fertility if they continued in the Ovsynch protocol (<10%), and therefore are resynchronized with an Ovsynch + P4 protocol to receive TAI at d42 after previous AI (15–20% of non-pregnant cows). By eliminating non-synchronized cows (no CL at NPD) and by using two doses of PGF in the protocol, the P/AI can be increased by 10–15%, thereby producing a higher fertility Resynch program.

## 5. Key Factors That Alter Reproductive Efficiency in Dairy Herds

**Figure 6:**
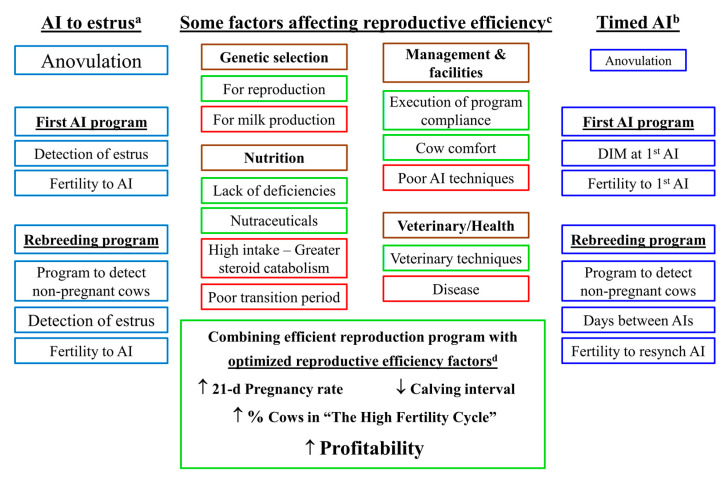
Representation of the key reproductive factors that directly affect a reproductive management program using artificial insemination (AI) to estrus (**left ^a^**) or timed AI (**right ^b^**). Some of the key factors that affect reproductive efficiency in either or both types of programs are shown (**center ^c^**). Factors shown in red squares tend to decrease fertility, whereas factors shown in green rectangles tend to increase fertility. (**Bottom ^d^**) Shows that implementation of effective reproductive management programs, combined with optimization of factors that alter reproductive efficiency allows farms to reach the goal of improved reproductive efficiency and profitability on a high-producing dairy farm. Days in milk (DIM).

As shown in Figure 6, there are multiple factors that determine the success of a reproductive management program. In herds using exclusively TAI programs (right side in purple), anovulation is less of a problem than in herds using exclusively AI to estrus (left side in blue). This is because TAI programs can induce cyclicity leading to AI in all cows, including anovular cows. In herds using TAI, the DIM at first AI can be chosen by the design of the program. In addition, herds using fertility programs such as double-Ovsynch can have high fertility at first TAI. The efficiency of the rebreeding program will depend on the timing of NPD and the design of the Resynch program. In contrast, herds that use AI to estrus are dependent upon cows returning to cyclicity (making anovulation a critical problem) and detection of estrus in these cows with proper timing of AI during all days of the week. Similarly, the rebreeding program is dependent upon detection of estrus. The fertility after AI to estrus may be more controlled by certain factors such as level of milk production than observed in TAI programs that more fully control the follicle size, length of dominance, and hormonal environment [5,6,99].

In the center section of Figure 6 is shown four categories (brown rectangles) of factors that can affect reproductive efficiency in herds that use TAI, AI to estrus, or a combination of the two methods in their reproductive management program. Some of these factors are expected to increase reproductive efficiency (shown in green rectangles), while other factors tend to decrease reproductive efficiency (shown in red rectangles). These factors are discussed in more depth in this section of the manuscript. At the bottom is shown the overall goals for optimizing all of these factors into an efficient reproductive management system so that there is an increase in 21 d-PR, decreased calving interval, increased percentage of cows in the high fertility cycle, and ultimately increased profitability for the dairy operation.

### 5.1. Genetic Selection for Health and Reproductive Traits

One key change in genetic selection during the last 20 years has been the shift to selection for reproductive and health traits rather than milk production alone [1,2,3,4]. This has been a key factor related to the increase in daughter pregnancy rate (DPR) that has been observed since 2000. The more dramatic increase in phenotypic DPR since 2000 compared to genotypic DPR indicates that management factors, along with selection for high fertility genetics, have played a key role in the improvement in phenotypic DPR, including development of systematic breeding programs using TAI [5,112]. A recent study reinforced the importance of genetics in reproductive performance by evaluating primiparous and multiparous cows based on their genomic DPR, using quartiles [113]. The herds used the same reproductive management program on all cows but found greatly improved reproductive performance using multiple measures (P/AI at first AI, number of services/pregnancies, percentage of cows pregnant at the end of lactation, and interval between calving and pregnancy) for cows in the top 25% for DPR as compared with the lowest 25%. For example, primiparous cows in the top quartile for DPR became pregnant 30 days earlier (165 vs. 195 d) and multiparous cows became pregnant 36 days earlier (140 vs. 176 d) as compared with cows in the lowest quartile [113]. Another recent study [114] randomized ~2400 primiparous cows by genetic merit for fertility (high, medium, and low) and to reproductive management strategy (TAI vs. primarily AI to estrus). Although fertility was greater for TAI (double-Ovsynch TAI) than for AI to estrus, the cows with the highest genetic merit for fertility had greater P/AI than the cows with lower genetic potential for fertility in either type of reproductive management strategy. Thus, selection of cows with high genetic potential for fertility is a strategy that can and should be utilized by all dairy herds regardless of whether they manage reproduction primarily with TAI or using estrus.

### 5.2. Optimizing Cow Comfort and Reducing Heat Stress

Many physiological, production and reproduction responses can be influenced by management and facilities, and in this section, we focus on heat stress, which has a tremendous negative impact on dairy operations, especially in tropical and subtropical regions. The negative impact of heat stress on reproduction is extensively reported in the literature, with the P/AI of hot seasons being 20 to 50% lower than cooler months of the year [115,116,117]. However, in addition to reproduction, the negative impacts of heat stress can also impact many other aspects of production, health, and profitability in dairy operations. For example, heat stress of dairy cows can influence dry matter intake [118], animal welfare [119], immune system and health [120], and have carryover effects into the next generation [121]. Effects of heat stress on reproduction can impact reproduction in the short term but also may have effects on oocyte quality for 40 to >100 days after the end of heat stress [122,123]. Lactating dairy cows under heat stress had reduced oocyte competence and quality, reduced fertilization rates, and poorer embryo quality [23,124].

Thus, heat abatement strategies are needed on all types of dairies, particularly, in areas with greater heat index and humidity. Some of the most common strategies for cooling cows are fans, shade, natural ventilation, and water-cooling-systems, such as misters and sprinklers. For instance, a study that compared shade with water-cooling systems, over 18 weeks, reported a greater efficiency of water-cooling systems in reducing rectal temperature and respiration rate [125]. In a study using cows on a pasture-based system, cooling for a short period of time (90 min before afternoon milking) or providing shade reduced body temperature and respiration rate compared to control cows; however, combining shade with sprinklers was the most efficient system during days with more intense heat stress (temperature-humidity index ≥69) [126].

The duration and location for cooling cows can vary, with some herds cooling cows in the holding pen, the feed line, or both. Using data from Israel dairy herds [127], cows not cooled compared to those cooled for 7.5 h/d (holding pen + feed line), or 4.5 h/d (only holding pen) had a greater decrease in summer milk production (3.6, 1.6, and 0.6 kg/d, respectively), lower summer/winter production ratio (90.7, 96.1, and 98.5%, respectively), and lower P/AI at first AI of the summer (15, 34, and 34%, respectively).

In herds that are implementing strategies to cool the cows, there are different issues to consider including volume of water used, water size droplet, sprinkler flow rate, length of time spent dampening compared to drying during the cooling cycle, among others, which have been extensively discussed in the scientific literature [128,129,130,131].

One exciting aspect, which has been introduced and discussed in the past few years, is the benefit of minimizing heat stress of dry-pregnant cows during the dry period. [121] reported interesting results on the impact of cooling multiparous dams with shade, fans, and water soakers during the dry period on the performance of the daughters and granddaughters. In the study, there was greater culling before first calving and productive life was reduced in daughters from dams that were not cooled compared to dams that were cooled. In addition, more granddaughters were culled before first breeding from dams that were not cooled. In terms of milk production, daughters from heat-stressed dams produced less milk in the first, second, and third lactations (2.2, 2.3, and 6.5 kg/d, respectively), and the granddaughters produced 1.3 kg less milk/d in their first lactation. Thus, the results highlight the importance of cooling cows to optimize their own production and reproduction, but also to improve performance of their offspring.

Lastly, thinking about reproductive management strategies to improve performance during heat stress periods, an interesting manuscript reported that embryo transfer (ET) could be an alternative tool for improving fertility [117]. The fertility was reduced in hot seasons of the years using either TAI or ET; however, P/ET was notably greater than P/AI in hot seasons and had less variation throughout the year compared to P/AI [117]. Thus, use of ET can reduce the negative impact of heat stress on fertility, and this may be especially important in tropical regions.

### 5.3. Importance of the Transition Period on Subsequent Fertility

Another critical factor for optimizing fertility in dairy herds is related to the transition period, defined as the period from 21 days before calving to 21 days after calving. Issues during the transition period can impact health during the subsequent lactation [132,133] body condition score (BCS) change, and fertility [134,135]. These factors impact the likelihood for a cow becoming pregnant early in lactation and entering “the high fertility cycle” [16], as discussed in Section 6.

A healthier transition period is key to the profitability of a dairy herd, due to the usually high incidence of diseases and associated costs [133,136], high incidence of culling and mortality [137,138], and the impact on production and reproduction [133,135]. For example, cows that did not have diseases within the first 21 DIM reached the peak of milk production earlier, produced more milk at peak, and produced ~360 and ~703 more kg of milk in a 305-day period compared to cows with one or more than one disease, respectively (10,453 vs. 10,096 vs. 9750 kg) [133].

Return to cyclicity during the postpartum period is critical for the success of reproductive programs, and the nutrition and management during the transition period is a critical determinant of time to first ovulation. Anovulation at ~60 DIM can range from 5 to 45% in different dairy herds [139] and is greater in cows that have a greater incidence of health problems and cows losing excessive BCS during the postpartum period [133,135,140]. For example, one recent study by [140] evaluated 943 Holstein cows during the postpartum period and reported that, at 50 DIM, a 17.9% prevalence of anovulation in healthy cows (38.4% of the cows) as compared with 29.8% prevalence in cows with one disease (33.7% of the cows) and 39.5% prevalence in those that had more than one disease (27.9% of the cows). As in many dairy herds, most (~60%) cows had at least one health problem in this study.

The timing of return to cyclicity can influence the timing of first AI and also the fertility to the first AI. For example, if a farm uses AI after detection of estrus as the main reproductive strategy for first service, anovulatory cows will receive their first AI later in lactation and may also have lower fertility to first AI as compared with cows that had undergone more cycles prior to first AI. In herds that use TAI, the synchronization strategy may allow early first AI, but the fertility is lower in cows that initiate TAI protocols in the absence of a CL or in low P4 concentrations as illustrated in Figure 1.

Diseases also negatively impact fertility. [132] evaluated 5719 cows from seven herds and reported greater P/AI at first service in healthy cows compared to those having one or more than one disease in early lactation (51.4 vs. 43.3 vs. 34.7%, respectively). Pregnancy loss (PL) was also affected by diseases, with 8% of PL in healthy cows compared to 13.9% and 15.9% in cows with one and more than one disease [132].

A large study that evaluated ~5000 cows reported a long-term negative effect of diseases on reproductive performance [133]. Cows, up to 150 DIM, that had diseases during the first 21 DIM had lower P/AI in early lactation and when inseminated. Moreover, the PL of those cows that had diseases was elevated. The lower P/AI combined with the greater PL among cows with diseases resulted in fewer cows calving/AI from those inseminated, up to 200 DIM. In addition, days open was lower in healthy cows (133.5 d) compared to cows with one disease (145.5 d) or multiple diseases (157.2 d). The percentage of cows calving again in the farm was greater for healthy cows than those with one or multiples diseases (72.8 vs. 59.6 vs. 47.3%, respectively). Therefore, these results show a clear and important impact of diseases during the transition period on early lactation and first AI, and a long-term negative impact on P/AI and PL, affecting the reproductive efficiency of cows during the entire lactation.

Continuing the discussion on the importance of the transition period, several studies have evaluated the physiology of energy and BCS changes of high-producing dairy cows, during the transition period and beginning of lactation. Two of those studies [134,135], discussed below, had a similar experimental design that focused on evaluations of cows that lost, maintained, or gained BCS during the transition period.

One initial interesting result is that only 50% or fewer cows lost BCS during the transition period. The percentage of cows that gained, maintained, or lost BCS during the transition period was 22.4%, 35.8%, and 41.8% in one of the studies [134] and 28%, 22% and 50% of cows in the other study [135]. In both studies, the BCS in the prepartum or at calving was lower for cows gaining BCS compared to cows that lost BCS in the transition period (2.57 vs. 2.97 [135] and 2.85 vs. 2.93 [134]). These results show that not all high-producing dairy cows lose BCS after calving and that cows with BCS of 3.0 or greater were the most likely to lose BCS.

Barletta et al. [135] reported that postpartum cows with increasing BCSs resumed cyclicity earlier than cows with decreasing BCSs (33.9 vs. 47.1 d), and that 100% of the cows with increasing BCSs as compared with 81.1% of the cows with decreasing BCSs were cyclic around 50 DIM. Moreover, the percentage of cows with more than one disease in early lactation was lower in cows gaining BCS compared to cows losing BCS (39.4 vs. 62.9%). The P/AI at first service was approximately three times greater for cows that gained BCS than cows that lost BCS during the transition period (53.9 vs. 18.3%). In the study by [134], with 1887 Holstein cows, the P/AI at first FTAI postpartum was 25%, 38%, and 84% for cows that lost, maintained, or gained BCS during the postpartum period. Therefore, these studies indicate that cows gaining BCS during the transition period have better health and cyclicity status in early lactation, and excellent fertility in the first service compared to cows losing BCS.

In summary, to optimize the transition period and reduce postpartum BCS loss, it is critical that cows calve with a relatively low BCS. On the one hand, overconditioned cows at calving (≥3.25) will have greater BCS loss, more health problems, later resumption of cyclicity, and lower fertility. Nevertheless, it is not advantageous for overconditioned cows to lose BCS during the dry period in an attempt to reduce BCS at calving. Loss of BCS during the dry period was associated with greater incidence of disease and lower fertility after calving [141]. On the other hand, underconditioned cows at calving (≤2.5) will have much later return to cyclicity, greater health problems, and lower fertility. In addition, other nutritional and management strategies can be implemented to prevent or decrease BCS loss and health problems in early lactation and this will improve performance and health of dairy cows and increase the likelihood of early pregnancy.

### 5.4. Nutritional Strategies to Optimize Reproductive Performance

Diets for lactating cows should be balanced to provide the required nutrients for milk production and for the reproductive process. Any deficiencies, whether in required vitamins, minerals, or nutrients, could produce nutritional conditions that might compromise reproduction. Nevertheless, provision of nutritional components in excess of requirements are not likely to lead to increased reproductive efficiency. This was clearly demonstrated by overfeeding of phosphorus (P) in dairy cattle diets during the 1980s and 1990s. On the basis of early studies that suggested cattle maintained on P deficient pastures had decreased calf crop, prolonged periods of anestrus, and poor reproductive performance, many nutritionists recommended feeding P in excess of requirements to improve reproductive performance. However, when we evaluated cows fed two levels of P (0.37%, recommended and 0.57%, excess), we found no differences in any measure of reproduction including return to cyclicity, expression of estrus, length of estrus, P/AI, or time to pregnancy [142,143,144]. Thus, although deficiencies of many nutrients may reduce reproductive performance, supplementing nutrients in excess of requirements may be expensive and is unlikely to improve fertility.

Nutrition during the dry period can have important implications for subsequent reproductive performance. A retrospective analysis of seven studies that compared higher energy diets and controlled energy diets (higher fiber) found that cows fed higher energy diets during the prepartum period had lower DIM during the postpartum period, greater BCS loss, and increased days open [145]. Thus, higher energy diets during the dry period should not be recommended. In some herds, vitamin E may be limiting during the dry period. In a study from our lab, dry dairy cows were receiving supplementation with less than the dietary recommendation for vitamin E and were randomized to either receive no treatment or to be treated weekly with three injections of 1000 IU each of DL-α-tocopherol administered during the last 3 prepartum weeks [146]. Vitamin E supplementation reduced the incidence of retained placenta (13.5 vs. 20.1%) and stillbirth (6.8 vs. 14.9%). Additionally, after first postpartum AI, for cows receiving vitamin E, pregnancy loss was reduced (12.5 vs. 20.5%) and considering all inseminations up to 200 DIM, Ps/AIs on Day 30 (38.4 vs. 34.5%) and Day 60 (32.8 vs. 26.9%) were greater (*p* < 0.05) and the pregnancy loss was lower (14.5 vs. 21.5%) as compared with cows who did not receive vitamin E. In addition, cows treated with the prepartum vitamin E injections had reduced days open (126 vs. 137 d). Thus, delivery of nutrients during the dry period, including vitamin E, need to meet requirements or there can be fairly severe consequences near calving with increased stillbirth and retained placenta, and subsequent reduction in reproductive performance. In this study, cows supplemented with vitamin E did not have increased milk production suggesting that the requirement for vitamin E, and possibly other nutrients, may differ for reproductive traits compared to production traits.

Hypocalcemia is another condition that is tied to the nutritional program during the transition period and can have important consequences for subsequent reproductive performance. In U.S. dairy herds, clinical hypocalcemia (<1.4 mM total calcium concentration) occurs in 5–10% of dairy cows, with subclinical hypocalcemia (1.4–2.0 mM total calcium concentration) occurring in an additional 50% of dairy cows [147]. Hypocalcemia in dairy cows is considered to be a gateway to disorders of the immune system and metabolism, leading to health and metabolic issues in periparturient dairy cows. The focus of recent research has been on the association of hypocalcemia with neutrophil function and development of metritis [148]. Recent studies have reported that cows developing clinical or subclinical hypocalcemia had decreased dry matter intake, greater negative energy balance, impaired immune function, increased risk of health problems, increased risk of metritis, decreased neutrophil number and activity, and decreased fertility and reproductive performance [147,148,149,150].

One of the most common and efficient strategy to reduce incidence of hypocalcemia is the use of acidogenic diets during the prepartum period. A recent meta-analysis [151] showed that a reduction in the dietary cation-anion difference (DCAD) in the prepartum period increased Ca concentration in the peripartum period in both primiparous and multiparous cows and increased milk production. The same meta-analysis reported that decreased DCAD in the diets reduced retained placenta and metritis and reduced health problems per cow in both multiparous and primiparous [151]. The DCAD does not need to be lower than −150 mEq/Kg of dry matter [151]; however, it is important to monitor the efficiency of the strategy by measuring urine pH in prepartum cows, to determine if the diet is inducing metabolic acidosis. Thus, using negative DCAD diets during the prepartum period is the most scientifically justified strategy for reducing hypocalcemia in dairy cattle.

Another nutritional strategy that has been evaluated for improving health, production, and reproduction is the inclusion of choline and methionine in the diets of lactating dairy cows. It is reported that increasing rumen-protected choline (RPC) in the prepartum diet increased pre- and postpartum DIM and milk production [152]. Moreover, RPC in the prepartum diet has been reported to decrease inflammation pre and postpartum and improved immune function, as evidenced by a greater proportion of neutrophils undergoing phagocytosis and oxidative burst in the postpartum period [153]. Feeding RPC to cows during pre and postpartum periods reduced the incidence of clinical ketosis, mastitis, and morbidity; however, in primiparous, RPC in the prepartum increased cases of fever and metritis [154]. Another nutrient that has been evaluated for effects on reproduction is feeding rumen-protected methionine (RPM). There is a consistent increase in milk protein percentage and protein yield by feeding RPM. In addition, methionine concentrations appear to be associated with optimal early embryonic development [144,155,156,157], and modulation of gene expression in early bovine embryos [156]. In addition, [158] fed RPM from 30 DIM until d60 of pregnancy and found that embryo size was increased in multiparous cows supplemented with RPM, as evidenced by greater increased embryonic abdominal diameter and volume and amniotic vesicle volume. There was no significant effect of RPM on P/AI, however, pregnancy loss was decreased by RPM feeding in multiparous cows [158]. In addition, feeding polyunsaturated fatty acids (PUFA) has been found to increase fertility in some experiments. For example, a study [159] with more than 700 dairy cows fed marine algae that was rich in PUFA, i.e., docosahexaenoic acid, daily from 27 to 147 DIM, reported increased P/AI in both primiparous and multiparous cows (41.6 vs. 30.7%) and a reduction in days to pregnancy by 22 d (102 vs. 124 d). Thus, RPC, RPM, and PUFAs may be considered to be “nutraceuticals” that can improve reproductive performance when included in diets of lactating dairy cows.

## 6. Implementation of Efficient Reproductive Management Programs: Achieving the High Fertility Cycle

On the basis of the data discussed above related to BCS loss and fertility [134,135], the research group of Richard Pursley at Michigan State University introduced the concept of “the high fertility cycle” [16]. They experimentally explored this idea in a study of 851 lactating Holstein cows (primiparous and multiparous), with an average milk production of 42 kg per d. Cows that had a calving interval (CI) of 13 months (~395 d) had lower BCS at parturition (≤2.7) as compared with cows with greater CI. Moreover, all cows with a 13-month CI maintained or gained BCS after calving [16]. Conversely, all cows with CI > 14 months had BCS ≥ 3.1 at parturition and all of them lost 0.5 or more points of BCS after calving [16].

Following a similar pattern as shown previously [134,135], the authors observed fewer health issues (7%) in cows that maintained or gained BCS as compared with those losing ≥0.5 points of BCS (30%). In addition, P/AI was greater (50.0 vs. 39.9%) and PL was lower (0.0 vs. 8.3%) for cows maintaining or gaining BCS as compared with those that lost BCS during postpartum [16].

Thus, these results are consistent with the concept that cows that become pregnant earlier postpartum (prior to 130–150 DIM), in other words, achieving a CI close to 13 months, it is likely that they will not be overconditioned at the time of calving, and therefore will have reduced BCS loss after calving and have fewer health issues. Of special importance to this review, those cows will have improved fertility and reduced pregnancy loss, allowing them to become pregnant earlier in lactation and thereby entering the high fertility cycle.

**Figure 7:**
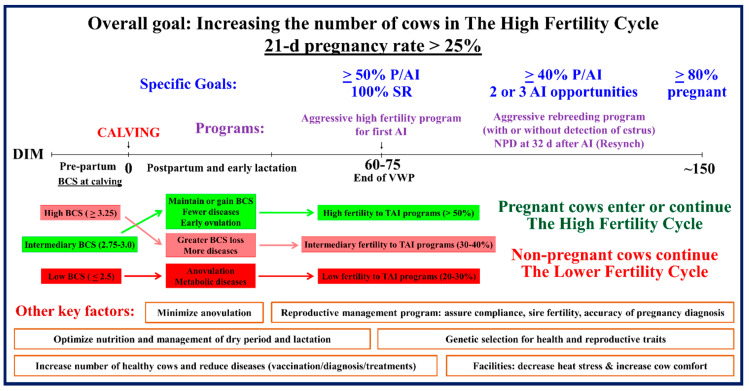
Concluding figure on how to increase the number of cows entering “the high fertility cycle”, including physiological aspects, reproductive management strategies, and factors that impact reproductive performance. Body condition score (BCS), voluntary waiting period (VWP), timed AI (TAI), nonpregnancy diagnosis (NPD), days in milk (DIM), and pregnancy per AI (P/AI).

Figure 7 attempts to synthesize all of the ideas discussed in this manuscript into a concluding figure that illustrates how to optimize reproductive efficiency in dairy herds that use TAI. A key concept is that a systematic program is designed by the management team, and then carried out on a consistent basis each week. The overall goal is to achieve a 21 d-PR above 25% with over 80% of cows pregnant by 150 DIM, thus, thrusting most cows in the herd into the high fertility cycle.

The first thing to consider when designing this program is how to achieve a cow with the optimal physiology to respond to a TAI or estrus detection program with high fertility. One of the critical factors to consider is achieving an intermediate BCS of 2.7–3.0 in cows as they approach calving. The nutrition and management programs need to be optimized to have excellent nutrition, health, and cow comfort, thereby reducing anovulation and increasing likelihood of cows achieving high fertility. As shown in Figure 7, the herd health program should be efficient in preventing, diagnosing, and treating diseases in order to maximize number of healthy cows that enter the first AI program.

The reproductive program used at first AI is critical for assuring the proper CI for the majority of cows. In most dairy farms, it would be advantageous to use a high fertility TAI program at first AI. This type of program can produce more than 50% pregnancies at first AI, with all cows receiving first AI at the end of the VWP. These fertility programs have been developed based on optimization of the physiology that produces high fertility. This includes a presynchronization strategy to induce cyclicity and assure that cows are at the proper reproductive state during the breeding protocols (Figure 4), promoting superior fertility as compared with strategies that inseminate cow based on standing estrus [6,99]. Nevertheless, some of the key cow factors prior to first AI, such as BCS loss, can produce subfertility results even to optimized TAI programs. In addition, other key reproductive factors must be considered, such as fertility of sires and use of proper AI technique, including thawing of semen, when suboptimal results are obtained in herds using fertility protocols at first AI, such as double-Ovsynch.

When cows do not become pregnant at first service postpartum, they need to be rapidly identified as nonpregnant and reinseminated. Therefore, to shorten the interbreeding interval, herds can detect estrus for second and greater services, although the success of this strategy will rely on the efficiency of the herd in detecting estrus and on the fertility of the cows inseminated in estrus. Alternatively, insemination of nonpregnant cows can be performed after resynchronization of ovulation using TAI protocols (commonly termed Resynch), usually initiated at the time of, or before, the NPD (Figure 5 and Figure 6) [40,107,111]. The 75 to 90 d from the first TAI to 150 DIM are critical; rebreeding strategies should be aggressive and optimized to achieve at least a two- or three-times higher fertility rate of AIs prior to 150 DIM. Rebreeding strategies that achieve 40% or more P/AI should ensure over 80% of cows are pregnant by 150 DIM. Programs should be designed in which fewer cows become pregnant after 150 DIM because these cows are likely to have excessive BCS at the next calving and are likely to not achieve the high fertility cycle.

Tools are available to dairy producers and veterinarians to achieve the high fertility cycle in high producing dairy herds. Some of these tools, such as optimized TAI protocols, have been extensively discussed in this manuscript and should be readily implemented in a dairy herd. Other tools for detection of estrus, such as activity monitors, tail-paint, and estrus detection patches are also available for herds that want to increase efficiency of estrus detection. These tools are likely to increase service rate but are unlikely to change P/AI directly, since they only allow more accurate detection of cows in estrus but do not change the underlying reproductive physiology. A more holistic or complete view of the reproductive management program also needs to be achieved by the management team in charge of optimizing reproduction. Many important nutritional, health, and facility details may be overlooked for their critical role in driving reproductive efficiency. The successful team should utilize the tools that match their reproductive management strategy and attempt to optimize all aspects of the program. The final detail that is important is: consistency, consistency, consistency.

## Figures and Tables

**Table 1:** Percentage of lactating dairy cows with a corpus luteum at the time of prostaglandin F2α (PGF) treatment comparing timed artificial insemination (TAI) protocols that utilized only estradiol benzoate (EB) vs. protocols with gonadotropin releasing-hormone (GnRH) either alone or combined with EB.

Study	*n*	EB on d0 of the TAI Protocol	GnRH on d0 of the TAI Protocol	Difference ^1^	*p*-Value
[67] Pereira et al. (2013) ^2^	1190	43.3%	72.5%	29.1%	<0.01
[68] Pereira et al. (2015) ^3^	1474	55.4%	69.5%	14.1%	<0.01
[29] Melo et al. (2016) ^3^	417	57.3%	76.4%	19.0%	<0.01
[66] Consentini et al. (2020) ^3^	369	56.6%	89.8%	33.2%	<0.01
Total	3450	52.0%	74.3%	22.2%	<0.001

^1^ The difference is in absolute percentage points. ^2^ Comparison between EB vs. EB plus GnRH on d0 of FTAI protocols. ^3^ Comparison between EB vs. GnRH on d0 of FTAI protocols.

**Table 2:** Pregnancy per AI (P/AI) of lactating dairy cows submitted to timed artificial insemination (TAI) protocols initiating with estradiol benzoate (EB), gonadotropin releasing-hormone (GnRH), or including a GnRH at the beginning. In all treatments, a progesterone (P4) implant was inserted on d0.

Study	*n*	Only EB on d0	GnRH on d0	Difference ^1^	*p*-Value
[68] Pereira et al. (2015)	1808	30.7%	36.8%	5.9% (19.2%)	<0.05 ^2^
[29] Melo et al. (2016)	1035	33.7%	38.2%	4.5% (13.4%)	0.07 ^3^
[76] Carneiro et al. (2017)	871	28.7%	38.2%	9.5% (33.1%)	<0.05 ^4^
		28.7%	34.5%	5.8% (20.2%)	0.10 ^2^
[66] Consentini et al. (2020)	943	37.5%	42.8%	5.3% (14.1%)	NS ^3^
		37.5%	42.0%	4.5% (12.0%)	NS ^5^
		37.5%	44.3%	6.8% (18.1%)	<0.05 ^4^
Total	4657	33.5%	39.5%	6.1% (18.2%)	<0.05

^1^ The difference is in absolute percentage points (relative % increase and difference/only EB). ^2^ Comparison between EB vs. EB plus GnRH on d0 of TAI protocols. ^3^ Comparison between EB vs. GnRH on d0 of TAI protocols. ^4^ Comparison between EB vs. EB on d0 plus GnRH on d2 of TAI protocols. ^5^ Comparison between EB vs. GnRH on d0 and d2 of TAI protocols.

**Table 3:** Effect of an additional treatment with prostaglandin F2α (PGF) during the Ovsynch protocol on complete luteolysis at the end of the protocol and on pregnancy per artificial insemination (P/AI).

Item	1 PGF during Ovsynch	2 PGF during Ovsynch	Range	*p*-Value
Complete luteolysis at the end of Ovsynch, % (*n*/*n*)	83.5 (788/944)	95.1 (867/912)	6–14	<0.001
P/AI, % (*n*/*n*)	34.0 (915/2689)	38.6 (1029/2667)	3–9	<0.001

Adapted from [82]. Meta-analysis with seven studies with randomized controlled designs.

**Table 4:** Pregnancy per artificial insemination (P/AI) 31 and 60 d after timed AI (TAI) and pregnancy loss according to the strategy to induce final ovulation. EC (estradiol cypionate 48 h before TAI), EC/G (estradiol cypionate 48 and GnRH 16 h before TAI), and G (GnRH 16 h before TAI).

Item	Strategy to Induce Final Ovulation in the TAI Protocol			*p*
	EC	EC/G	G	
P/AI on d31, % (*n*/*n*)	42.5 (99/233)	43.0 (95/221)	42.8 (89/208)	0.45
P/AI on d60, % (*n*/*n*)	37.1 (86/232)	37.5 (81/216)	33.7 (69/205)	0.42
Pregnancy loss, % (*n*/*n*)	12.2 (12/98) ^A^	10.0 (9/90) ^A^	19.8 (17/86) ^B^	0.09

^A,B^ Different letters represent differences (*p* < 0.05). When the two groups that received EC (EC and EC/G) were combined, there was lower pregnancy loss compared to cows receiving only GnRH [11.2 (21/188) vs. 19.8 (17/86), *p* = 0.05). Adapted from [86].

**Table 5:** Effect of Double-Ovsynch or management aimed to inseminate cows in estrus on submission rate, pregnancies per artificial insemination (P/AI) 33 and 63 d after insemination, and percentage of pregnant cows at 33 and 63 d after first insemination in lactating Holstein cows with similar days in milk.

Item	Strategy for First AI		Difference, % (*p*-Value)
	Double-Ovsynch	Estrus	
No. of cows	294	284	
Submission rate, % (*n*/*n*)	100.0 (294/294)	77.5 (220/284)	29 (<0.01)
P/AI at 33 d, % (*n*/*n*)	49.0 (144/294)	38.6 (85/220)	27 (002)
Pregnant cows at 33 d, % (*n*/*n*)	49.0 (144/294)	29.9 (85/284)	64 (<0.01)
P/AI at 63 d, % (*n*/*n*)	44.6 (131/294)	36.4 (80/220)	23 (0.05)
Pregnant cows at 63 d, % (*n*/*n*)	44.6 (131/294)	28.2 (80/284)	58 (<0.01)

Adapted from [99].
